# Zearalenone and the Immune Response

**DOI:** 10.3390/toxins13040248

**Published:** 2021-03-31

**Authors:** Cristina Valeria Bulgaru, Daniela Eliza Marin, Gina Cecilia Pistol, Ionelia Taranu

**Affiliations:** 1 Laboratory of Animal Biology, National Institute of Research and Development for Biology and Animal Nutrition, 077015 Balotesti, Romania; cristina.bulgaru@ibna.ro (C.V.B.); gina.pistol@ibna.ro (G.C.P.); 2 Faculty of Biology, University of Bucharest, Splaiul Independentei 91-95, R-050095 Bucharest, Romania

**Keywords:** zearalenone, metabolites, innate immunity, cell immunity, humoral immunity

## Abstract

Zearalenone (ZEA) is an estrogenic fusariotoxin, being classified as a phytoestrogen, or as a mycoestrogen. ZEA and its metabolites are able to bind to estrogen receptors, 17β-estradiol specific receptors, leading to reproductive disorders which include low fertility, abnormal fetal development, reduced litter size and modification at the level of reproductive hormones especially in female pigs. ZEA has also significant effects on immune response with immunostimulatory or immunosuppressive results. This review presents the effects of ZEA and its derivatives on all levels of the immune response such as innate immunity with its principal component inflammatory response as well as the acquired immunity with two components, humoral and cellular immune response. The mechanisms involved by ZEA in triggering its effects are addressed. The review cited more than 150 publications and discuss the results obtained from in vitro and in vivo experiments exploring the immunotoxicity produced by ZEA on different type of immune cells (phagocytes related to innate immunity and lymphocytes related to acquired immunity) as well as on immune organs. The review indicates that despite the increasing number of studies analyzing the mechanisms used by ZEA to modulate the immune response the available data are unsubstantial and needs further works.

## 1. Introduction

Fusariotoxins are secondary metabolites originate from fungi of the *Fusarium* and *Gibberella* species which represent the largest group of mycotoxins (more than 140). Of these the most widespread and also of primary concern are the trichothecenes, fumonisins, and zearalenone [1,2]. Despite the mitigation efforts, exposure of crops to mycotoxins is indeed inevitable and decontamination is very difficult [3,4]. Even though numerous studies concerning the effect of mycotoxins are reported in the specific literature, the contamination of cereals with fusariotoxins, the impact on animal health and the economic losses they imply [5,6,7], the transmission of fusariotoxins into the target organism and the potential existence of toxic components in meat and dairy products remain unknown and need further investigations [8].

**Figure 1:**
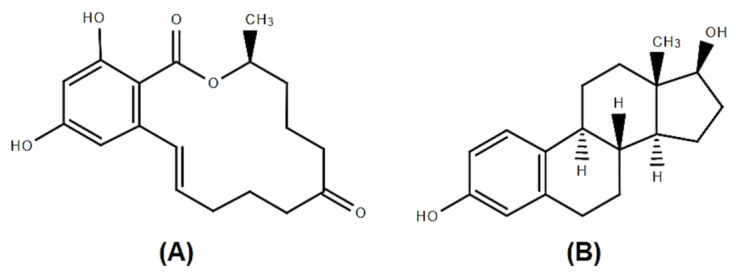
Chemical structures of Zearalenone (ZEA) (**A**) and 17β-estradiol (**B**).

Zearalenone (ZEA) is a fusariotoxin that belongs to the class of xenoestrogens due to its structural similarity to 17β-estradiol (Figure 1), but also due to the binding affinity to estrogen receptors [9]. It is a resorcyclic acid lactone [10], produced by *Fusarium graminearum, Fusarium cerealis, Fusarium semitectum, Fusarium culmorum and Fusarium equiseti* [11] which binds to estrogen receptors in mammalians target cells leading to reproductive disorders especially in female pigs [12]. In terms of physicochemical properties, ZEA has been shown to have high stability, being resistant to high temperatures and UV radiation, making it almost impossible to decontaminate crops on a large scale [13].

ZEA is metabolized mainly at the intestinal and hepatic level and transformed in several metabolites by hydroxylation, glucuronidation or conjugation reactions [14]. Biotransformation of ZEA lead primarily to the formation of two metabolites, α-zearalenol (α-ZOL) and β-zearalenol (β-ZOL) which can be further reduced to α-zearalanol (α-ZAL) and β-zearalanol (β-ZAL) [15].

After oral contamination, zearalenone is rapidly absorbed in mammals (rabbits, rats, humans and pigs, 80–85%) [16,17]. As mentioned above, structurally, ZEA has a strong similarity with 17 β-estradiol, and is able to bind to estrogen receptors, being also classified as a phytoestrogen, or as a mycoestrogen [18]. From this reason ZEA intoxication most often leads to disorders of the reproductive system. The changes involve low fertility, abnormal fetal development, reduce litter size and modification in the level of specific reproductive hormones: Estradiol and progesterone [19]. But, ZEA manifests its toxicity on many other systems besides the reproductive one. Studies show that the liver and spleen are also affected when exposed to this mycotoxin. It has been demonstrated in an experiment on female piglets that excessive amounts of ZEA raises the key liver enzymes level such as glutathione peroxidase and decreases spleen weight [20]. ZEA exerts significant effects on immune response, the major defense mechanism against pathogens, toxins and other antigens in all mammals with immunostimulatory or immunosuppressive results [21]. The cellular mechanisms activated by zearalenone in triggering its different effects are not yet well understood.

This review presents the effect of ZEA and its derivatives on all levels of the immune response such as innate immunity with its principal component inflammatory response as well as the acquired immunity with two components, humoral and cellular immune response. The mechanisms involved by ZEA in triggering its effects are also addressed in this review. The gut is the first organ exposed to mycotoxins, after oral contamination [22]. For this reason, the review presents initially the effect of ZEA on the gut.

## 2. Effect of ZEA on Gut Immunity

The gut represents a physical barrier that ensure the body’s protection against environmental agents, mycotoxins included [23]. This barrier involves intestinal epithelial cells (IECs), but also components of both innate and adaptive immunity, as IgA and pro-inflammatory cytokines or molecules involved in the inhibition of bacterial colonization (mucins and antimicrobial peptides) [24].

A recent study of Lahjouji et al. [25] reported that the intestine is a target of ZEA which cause pathological intestinal changes. Depending on different factors as specie, sex, dose or time of exposure, ZEA can affect or not the normal anatomic structure of the intestine. For example, ZEA (40 μg/kg b.w.) did not change the mucosa thickness, the height of villi or the number of goblet cells in swine [26,27], but the ingestion of ZEA (0.3–146 mg/kg feed) in pregnant rats modified the structure of villi and decrease the junction proteins expression [28]. Also, in the first week of a chronic exposure of pigs to ZEA (40 &micro;g/kg b.w.) produced transitory morphological modifications of small intestine and an increase in Paneth cell numbers in the crypt [25].

Exposure to ZEA was associated with the impairment of cell viability, apoptosis and necrosis in different organs including gut [29,30]. For example, recent studies have shown that treatment with ZEA at 10–100 µM for 24h decreased viability of porcine intestinal epithelial cells IPEC-J2 [31] and IPEC-1 [32,33] and increase LDH activity [31]. Apoptosis is one of the main mechanisms involved by ZEA induced toxicity in the gut and it was shown that ZEA can cause apoptosis in various intestinal epithelial cells as IPEC-J2 [34], MODE-K [35] and HCT116 [36]. Sub-chronic exposure of female Polish Large White pigs to low doses of ZEA increased apoptosis and decreased the ileal Peyer’s patches lymphocytes proliferation [37].

Some recent studies have shown that ZEA exposure alters or not the T- and B-cell subtype populations in small intestine. The administration of ZEA (5 or 20 mg/kg b.w.) to female BALB/C mice increased the percentages of CD8+ and decreased the percentages of CD4+ cells in the lamina propria and intra-epithelium lymphocytes, while no effect on CD8+ and CD19+ lymphocytes was observed in the Peyer’ patches [38]. Also, the exposure of prepubertal gilts to 100mg ZEA/kg feed decreased the percentage of CD21+ lymphocytes in pig ileum [37].

Endoplasmic reticulum stress (ERS) pathway seems to be the main signaling pathway involved in ZEA induced apoptosis. In MODE-K mouse cells exposed to zearalenone, the toxin increased the gene expression and protein synthesis of molecules involved in ERS-induced apoptosis pathway as: c-Jun N-terminal kinase, C/EBP homologous protein, GRP78, caspase-1 and the anti-apoptotic protein Bax, while decreasing the levels of the pro-apoptotic related protein Bcl-2 [35]. Similarly, an increased number of apoptotic cells were observed in the Peyer’ patches of the 20 mg/kg ZEA-treated mice associated with a significantly increase of Bax gene expression and of the ratio of Bax:Bcl-2 [38].

Mucosal IgA protects the intestinal epithelium from xenobiotics, viruses and bacteria and maintains also the homeostasis of the intestinal environment [39]. Indeed, previous studies have linked the increase of IgAs to repeated exposure to environmental toxins or to chronic infections [40]. The effect of ZEA on IgAs synthesis is controversial. ZEA administered by oral gavage to female BALB/c mice for two weeks at a dose of 5 or 20 mg/kg b.w. provokes a significantly decrease of the mucosal IgA antibody level in mice duodenum [38]. On the other side, the administration of 20 mg of ZEA/kg b.w. for only one week to male BALB/c mice significantly increased fecal IgA levels in the jejunum (1.5-fold higher that in the control group) [41]. Because the same concentration of ZEA (20 mg/kg b.w.) and the same way of administration (oral gavage) were used in both experiments, the different results could be related to the experiment duration (one week vs. two weeks) or could be influenced by the sex of the animals (male vs. female) and the intestinal segment.

Mucin glycoproteins are produced by epithelial or submucosal mucus-producing cells and represents the principal constituent of mucus [42]. The mucus structure is permanently renewed and could be rapidly adjusted to respond to the changes of the environment, as for example in response to microbial infection or to exposure to contaminants [30]. Recent studies have shown that ZEA (20 mg/kg b.w.) is responsible for intestinal mucosa abnormalities [41] as mice exposure for a short period (one week) to ZEA significantly increased the expression of mucin 1 and mucin 2 genes in the gut. Results on in vitro cell cultures also indicates that ZEA interferes with the mucin synthesis since the toxin increased the concentration of these molecules in Caco-2/HT29-MTX cells [43]. In vitro exposure of swine IPEC-J2 cells to 40 µM ZEA [44], and in vivo exposure of mice to 20 mg ZEA/kg b.w. [41] resulted in the upregulation of β-defensin, an important antimicrobial peptide, with important consequences for the defense against intestinal infection.

The effect of ZEA on the intestinal inflammatory response is not very clear, and this fact is due probably to the different experimental models and various ZEA concentrations used in several studies. Thus, in vitro studies showed that the incubation of porcine intestinal cells with 10 and 40 µM of zearalenone respectively lead to the increased expression of genes encoding for molecules involved in inflammation such as TLRs and the cytokines: *IL-1β, TNF-α, IL-6, IL-8, IL-12p40* in IPEC-1 [22] and of *IL-1β* and *TNF-α* in IPEC-J2 cells [44]. The increase in the expression of pro-inflammatory cytokines (IL-1β and TNF-α) was also registered in the in vivo studies, in jejunum of mice intoxicated with 20 mg ZEA/kg b.w. Also, prepubertal gilts exposed to 100 mg ZEA/kg feed for 42 days had elevated ileum concentrations of IL-12/23 40p and IL-1β [37]. By contrast, other researchers found that ZEA had no effect on proinflammatory IL-8 cytokine synthesis by IPEC-1 cells [32] or decreased the gene expression of *IL-1β*, *TNF-α* and *IL-8* in the jejunum of rats when administered in different doses (0.3–146.0 mg/kg feed) [28]. As well, a microarray study performed on IPEC-1 cells exposed to ZEA (25 μM) found a decrease of the expression of pro-inflammatory cytokines *IL-8*, *TNF-α* and *IL-6* [45]. The inflammatory effect produced by ZEA can be transmitted from mother to offspring. Piglets derived from sows fed 300 ppb zearalenone one week before farrowing and during the lactation period developed gut inflammation [46].

## 3. Effect of ZEA on Innate Immune Response

Innate or nonspecific immunity is the first form of defense for multicellular organisms. The innate immune response is triggered by receptors that recognize the pathogen and activate a series of signaling pathways that control the immune response [47]. Neutrophils, NK and NKT cells, monocytes/macrophages and dendritic cells that mediate interactions with pathogens are the innate immune system components able to form networks with key role in the initial immune response to infection and tissue damages [48]. They are phagocytic cells that when stimulated can produce reactive oxygen species (ROS), important in cell signaling and homeostasis [49,50]. An imbalance between ROS production and its inefficient elimination drives to a dramatic increase in ROS levels leading to cells damage, known as oxidative stress [49]. Recently, Wang et al., reported that ZEA (5, 10, 20 µM) increased ROS production in bovine neutrophils and decreased antioxidant enzymes (SOD and CAT) activity by involving NADPH, ERK and p38 activation followed by the formation of neutrophil extracellular traps (NETs), a network of DNA extracellular fibbers which help neutrophil cells to kill extracellular pathogens [51]. This could have significant cytotoxic and pro-inflammatory consequences.

According with the study of Marin et al., zearalenone and its metabolites decreased cells viability of porcine polymorphonuclear cells, interleukine-8 synthesis and increased superoxide production, ZEA metabolites being more immunotoxic than ZEA [52]. Moreover, Murata et al., found that the effect of ZEA and its metabolites on bovine neutrophiles depend on their chemical structure [53]. Thus, these authors found that zearalenone and its derivatives α- and β-zearalenol suppressed luminol dependent PMA chemiluminescence in neutrophils due to their C1’-2’ double bonds while, zearalanone, α- and β-zearalanols did not exert this effect because they possess a hydrogenated C1’-2’ bond instead of the double bond. In vivo experimentation conducted on Ross 308 hybrid broilers fed with two concentration of deoxynivalenol and zearalenone after hatching showed that the toxins induced oxidative stress and inhibited significantly the blood cells phagocytic activity [54]. In combination with other mycotoxins (alternariol, deoxynivalenol), ZEA affect innate immune functions by inhibited for example the differentiation of monocyte into macrophages (THP-1 cell line). Moreover, the combination of ZEA with alternariol at low concentration lead to synergistic effect on CD14 expression [55]. The combination of the two toxins altered the macrophages functions by the inhibition of TNF-α secretion. The primary function of RAW 264.7 macrophages such as proliferation was reduced and apoptosis was induced in a dose-dependent manner by zearalenone (from 0 to 100 µM) via the ERS pathway [56]. Three milligrams of ZEA /kg b.w. altered in in vivo treated rats the hydrogen peroxide release by peritoneal macrophages [57]. Also in vivo, the low concentrations of ZEA (40 μg/kg b.w. per day) alone or in combination with deoxynivalenol (DON, 12 μg/kg b.w. per day), another *Fusarium* mycotoxin, produced changes in the morphology of pig Kuppfer cells (stellate macrophages) with consequences on their activity [58].

Inflammation represents a rapidly nonspecific immune response through which the phagocytic cells are activated and produce bioactive molecules (inflammatory cytokines, prostaglandins and leukotrienes) as well as oxygen and nitrogen metabolites [59]. Being an agonist of the estrogenic receptors, ZEA can modulate similarly the in vitro and in vivo inflammatory response depending on its concentration, time of exposure and immune indices investigated.

In vitro studies highlight an increase or a decline of inflammatory response induced or not by ZEA depending on the immune cell type. Thus, the in vitro study of Marin et al., in which swine PBMCs were exposed to ZEA and its metabolites for 48h showed that ZEA decreased significantly at 5 and 10 µM the TNF-α response of these cells and had no significantly effect on IL-1β and IL-8, while zearalanone decreased also the production of IL-8 at 10 µM [19]. Interleukin 8 (IL-8) is a common inflammatory cytokine important in the recruitment of the immune cells, a key parameter in localized inflammation which induced after that an increase of oxidative stress mediators [60]. The same authors reported later [32] that ZEA derivatives, alpha-zearalenol (α-ZOL), beta-zearalenol (β-ZOL), and zearalanone (ZAN) decreased in a dose dependent manner the IL-8 synthesis in polymorphonuclear cells (PMN), significantly at 10 µM (−49.2% for α-ZOL; −45.6% for β-ZOL and −45.1% for ZAN respectively) after 3 h of exposure. No effect of ZEA on IL-8 concentration registered. Similarly, ZEA metabolites were more potent than ZEA itself in decreasing the IL-8 synthesis in porcine epithelial cells (IPEC-1) [32]. By contrast, Ding e al. [61] found that the level of IL-1β and IL-18 increased significantly in peritoneal mouse macrophages isolated from mice receiving ZEA by gavage (4.5 mg/kg b.w.) once a day for 9 days and treated in vitro with 8 µg/mL for 24h. In a model monocytic cell line, hER + IL-1β-CAT+, the exposure of the cells to low level 50 ng/mL of zearalenone and α-zearalenol resulted in a pro-inflammatory effect as 17β-estradiol by modulating and promoting IL-1β synthesis. The toxins manifested full agonist activity with 17β-estradiol but at lower potency [62] exposure of human placental choriocarcinoma (BeWo) cells to different concentrations of ZEA (2–16µM) increased the IL-6 production [63].

In vivo studies confirmed the biphasic effect of zearalenone on inflammation. Experiments performed on piglets found that in spleen and blood, ZEA increased the gene expression of pro-inflammatory *TNF-α, IL-6, IL-8* and *IL-1β*, while in liver the toxin decreased dramatically the expression of these cytokine which might have consequences on immune homeostasis taking into account that liver is considered a key organ for immune homeostasis [64]. It seems that the pro- or anti-inflammatory effect of ZEA depend on the organ involved. The earlier results of Salah-Abbes [65,66] showed a significant reduction of TNF-α, IL-1β and IL-12 in plasma of mice treated with 40 mg ZEA /kg b.w. as well as an increase of the cytokines (TNF-α, IL-6, IL-10) in kidney of mice fed the same concentration of toxin [67]. The inflammatory reaction in kidney is produced through the activation of macrophages which, once activated, will further produce inflammatory cytokines and chemokines subsequently responsible for different reactions and effects such as apoptosis and necrosis, the up- or down regulation of pro- and anti- apoptotic genes [67]. In testicular tissue of mice exposed for 48h to ZEA (40mg/kg b.w.) the toxin increased the level of TNF-α, IL-1β, IL-6 and decreased the level of anti-inflammatory IL-10 cytokine [68].

## 4. Effect of Zearalenone on Adaptive Immune Response

### 4.1. Effect of Zearalenone on Humoral Immune Response

Animal exposure to different doses of zearalenone has resulted in an alteration of humoral immunity as can be seen in Table 1. Literature studies show that ZEA leads to a decrease in serum IgG levels regardless the animal species (mice, rat or swine), toxin concentration or the duration of the exposure. Similarly, most studies have shown that the level of IgM in serum decreases no matter the species (mice or rats), time of exposure (12–36 days) and mycotoxin concentration (5–30 mg/kg b.w.). However, sub-chronic exposure (3–4 weeks) of rats to lower concentrations of ZEA (2–4 mg/kg b.w.) was associated with an increase in serum IgM concentration [69,70]. It seems that the effect of ZEA on IgM concentration is correlated with the sex of animal, since male piglets exposed to low concentration of toxin (0.8 mg/kg feed) resulted in a decreased in IgM level, while no significant changes were observed in serum IgM concentration of gilts receiving ZEA higher concentrations (1.1–3.2 mg/kg feed). Serum IgA concentration was not affected in mice, rats or swine after the exposure to low and medium concentration of the toxin (0.08–30 mg/kg feed) as resulted from most studies (Table 1) and it is not related to the time of exposure (18–42 days). BALB/c female mice fed higher concentration of ZEA (40 mg/kg feed) for 48h showed a decrease of IgA concentration [71].

Moreover, ZEA metabolites interfere with immunoglobulin synthesis. As it was shown in an in vitro study using swine peripheral blood mononuclear cells, both ZEA and its metabolites (α-ZOL, β-ZOL and ZAN) significantly decreased the immunoglobulins IgG, IgM and IgA synthesis at concentrations higher that 5 µM [19].

It was observed that the consumption of contaminated feed led to an increase of toxin concentration in the serum of intoxicated animals before farrowing and during lactation suggesting that ZEA or its metabolites can interfere with immunoglobulin secretion in colostrum/milk and in offspring. Also, α-zearalenol metabolite was found in the colostrum and milk of the sows [46], but in our knowledge no data concerning this interference are available until now in the literature. However, feeding sows with a dietary mixture of mycotoxins containing DON, ZEA and fusaric acid resulted in a decrease of the concentration of IgA in the colostrum and of IgA and IgG in serum of their offspring [78].

A common method for the assessment of the T-cell-dependent antibody responses is represented by the sheep red blood cells (SRBCs) assay. Few data (Table 2) concerning this type of immune response related to ZEA exposure are available. For example, exposure to 10 mg ZEA /kg b.w. of female B6C3F1 mice had no effect on the splenic plaque forming cells in response to SRBC [79] while a decrease of the B cells producing immunoglobulin M antibody to SRBC was observed in female Wistar rats exposed for 28 days to 3 mg of ZEA/kg b.w. [57].

In a recent review concerning the impact of *Fusarium* mycotoxins on human and animal host susceptibility to infectious diseases, it was shown that in contrast to other fusariotoxins, the interaction between zearalenone and infectious disease was less studied [80]. Pestka and collaborators showed that mice fed 10 mg ZEA/kg feed for 2 weeks and infected with *Listeria monocytogenes*, registered a decreased resistance to Listeria and an increase of the bacterial count in spleen as compared with control animals [79]. It can be claimed that ZEA exposure interferes with the capacity of organism to realize an adequate immune response to vaccination and that the toxin can alter the specific antibody synthesis. Indeed, several studies have shown a decrease of antibody titer to porcine parvovirus [72] or to swine plague [76] in zearalenone intoxicated animals, but however more studies are needed in order to better understand the relation between zearalenone and the response to infectious disease.

### 4.2. Effect of Zearalenone on Cellular Immune Response

Beside its effect on humoral immune response, ZEA cause negative effects on cellular immune response (e.g., cell viability and proliferation, apoptosis and necrosis, and cytokine production) due to the fact that most of the cells involved in the immune response have estrogenic receptors on their surface [82]. A disturbance of cell proliferation and apoptosis was reported in a number of studies investigating ZEA toxicity. As proved by many studies zearalenone is an inductor of apoptosis and necrosis in different type of immune cells. B and T lymphocytes are among the immune cells affected by the action of ZEA. It seems in fact that the immunosuppression produced by ZEA is caused by the decrease in B and T lymphocytes viability and proliferation [66,83]. These authors reported that ZEA (0.2–1800 ng/mL) produced a reduction of peripheral lymphocytes and this was a consequence of apoptosis and cell death triggered by ZEA at the spleen level knowing that spleen is one of the most important organs for maturing lymphocytes [66,71]. The death of spleen lymphocytes leads to the decrease of the peripheral lymphocytes. Zearalenone metabolites also reduced the lymphocytes proliferation. EFSA Scientific Opinion (2011) [84] cited the work of Forsell [85] in which the proliferation of human lymphocytes stimulated with different mitogens was reduced with 50% by 3.5 μg/mL zearalenone, 6.3 μg/mL α-zearalenol, 36 μg/mL β-zearalenol, 3.8 μg/mL α-zearalanol and 33 μg/mL β-zearalanol. The inhibition of proliferation was not related to the estrogenic potential of ZEA and its derivatives, but to their structure and the presence of a single or double bond. The presence in C-6’ of a keto parent molecule (ZEA) or alpha-hydroxyl substituents (alpha-ZEL and alpha-ZAL) led to a 10-fold higher toxicity [86]. Comparing the in vitro effect of zearalenone and is derivatives α-zearalenol and β-zearalenol with that of trichothecenes on proliferation of human peripheral mononuclear cells (PBMC) [83] observed that only the high concentration of these toxins had significant immunosuppressive effect. Indeed, in vitro investigation of Vlata et al. [87] on freshly human PBMC using increased concentration of zearalenone (0.1, 1, 5, 10, 30 µg/mL) showed that the highest concentration of ZEA (30 µg/mL) inhibited the proliferation of B and T lymphocytes and induced also a necrotic effect. A clear necrotic effect was also found irrespective of cells stimulation. The study of Zhang et al. [88] demonstrated that ZEA at 10–50 μg/mL had a time and dose dependent inhibitory action on mouse thymic epithelial cells proliferation and arrested thymic cells in G2/M phase of cellular cycle. Studies performed on TM3 cells shows that low doses of ZEA increases cell proliferation [89].

ZEA decrease not only the lymphocytes viability and proliferation but also lymphocytes phenotype number. A decreased expression of T (CD3+, CD4+, CD8+), NK and B lymphocytes was observed by Salah-Abbes et al. [66], when BALB c male mice were treated with ZEA 40 mg/kg. In the same line, Swamy et al. [90] pointed out that a diet naturally contaminated with *Fusarium* mycotoxins, ZEA (0.4 mg/kg and 0.7mg/kg) among them decreased linearly the number of B-cells, CD3+, CD4+, CD8+ lymphocytes and NK cells in broiler chickens via the reduction of interferon-β levels and IL-2 expression. Studies in mice, rats or pigs indicated a decrease in splenic coefficients, including proliferation and cell viability, the most affected cell populations being CD4+ and CD8+.

CDs (Clusters of Differentiation) are glycoproteins expressed on the surface of the immune cells. T cells are characterized by the expression of CD3, CD4 and CD8 markers [91,92] which are involved in in the transduction of signals from T cell surfaces (CD4 and CD8), while CD3 markers activate both the cytotoxic and helper T cells. As can be seen also in Table 3, there are conflicting data concerning the effect of ZEA on T cells subpopulations. While, most studies indicate a decrease in CD4+, CD8+ and CD3+ expression under the influence of ZEA, regardless of the animal species, other studies suggest an increase in CD4+ and CD8+ expression. However, any change in the CD4/CD8 ratio may indicate an immune dysfunction [91,92].

Induction of cellular death and proliferation inhibition was also found on other type of immune cells than lymphocytes. Viability of polymorphonuclear cells was decreased after 24h by 50 µM of ZEA and its metabolites α-ZOL, β-ZOL and ZAN action [52] and the exposure of RAW 264.7 macrophages to 10 to 100 μM ZEA for 24h diminished the cell viability in a dose dependent manner through apoptosis and necrosis [56,93].

By its estrogenic like-effects, ZEA impacts the development of reproductive organs irrespective of animal species, but with a different sensitivity of cells.

In weaned piglets, ZEA (0.5, 1.0 and 1.5 mg/kg) induces ovarian development by accelerating ovarian follicles proliferation through the activation of ERs/GSK-3β-dependent Wnt-1/β-catenin signaling pathway [94]. Also, ZEA (0.5–1.5 mg/kg) determined an abnormal uterine proliferation through TGF signaling pathway [95]. Investigating other regulatory pathways involved by ZEA in uterine hypertrophy, these authors exposed porcine endometrial epithelial cells to ZEA 0, 5, 20 and 80 μmol/L for 24 h and cell cycle was analyzed. A significant lower proportion of cells in S and G2 phases and an increase in the phase of G1 was found at ZEA 80 μmol/L [95]. The related mechanism involved also the activation of Wnt/β-catenin signaling pathway.

In mouse ovarian granulosa cells Chen et al. [96] and Zhang et al. [97] demonstrated by MTT, EdU and flow cytometry that ZEA suppressed in vitro cell viability at 30–150 μM and increased apoptosis at 15–60 μM after 24 or 72h of exposure. Close to the results recorded by Song et al. [95] in pig (a decreased of cell proportion in the S and G2 phase at ZEA 80 µmol/L), Zhang et al. [97], found that mouse granulosa cells were arrested in G1 phase of cell cycle and the cells proportion decreased in phase S and G2 after 30 μM ZEA treatment. However, in another study this author [98] found species specific ZEA effect, pig being more sensitive than mouse. Thus, ZEA 10 μM significantly increased the percentage of TUNEL porcine positive cells while the TUNEL percentage of granulosa mouse cells increased only at 30 μM.

Also, it has been observed that the metabolite of ZEA, α-ZOL at 9.4 µM concentration induces an increase in porcine granulosa cell proliferation and in progesterone levels [99].

In rats, ZEA perturb cell proliferation in both female and males. In Sprague Dawley males receiving by gavage 10 or 20 mg ZEA/kg b.w., the toxin significantly decreased the numbers of Leydig cells (adjacent cells to the testicle seminiferous tubules) which could produce anomalies of the male reproductive tract [100]. Similar results on Leydig cells were found by Wang et al. [101], with ZEA 50 μM. By contrast, Zheng et al. [89], found that low doses of zearalenone (0.01, 0.02, 0.03, 0.04, and 0.05 μmol/L) stimulated cell viability of TM3 cells (Leydig cells) measured by using the xCELLigence real-time cell analysis. Also, under the action of ZEA (20μM), cell viability of Sertoli cells derived from Male Wistar, which are important for male reproductive system, increased over control [102].

In human, study of Marton et al. [103], on ovarian epithelial cells investigating the effect of several compounds, ZEA among them on miRNA expression in correlation with cells estrogenic sensitivity observed that ZEA (1, 10, 100, 1000 nM) increased the rate of cell proliferation in direct proportion to ZEA concentration and depending on the presence of ER-α. By contrast, 30 µM of ZEA in prostate cancer cells induced G2/M cell cycle arrest and decreased cell viability compared to control [104].

Other examples concerning the effect of zearalenone on cellular immune response are illustrated in Table 3.

## 5. Effect of Zearalenone on Immune Organs

Zearalenone is responsible for the increase of reproductive organs’ weight such as the uterus [112]. By contrast, the weight of the immune organs seems to be less affected by the exposure to ZEA as resulted from the literature data (Table 4), but the toxin has been responsible for immune organs atrophy and depletion as well as for other histopathological modifications in immune organs. ZEA caused also a decrease of B cell percentage in the spleen or swelling of red pulp [57,108,111].

##  6.Mechanisms of Action

Zearalenone and its metabolites are considered to be endocrine disruptors, due to the similarity between their chemical structure and that of the endogenous estrogen, 17-estradiol, which allow them to bind to estrogen receptors (ERs) [119]. ZEA can bind to ER-α and ER-β [120], being a partial antagonist for ER-β and complete agonist for ER-α [121]. Among ZEA metabolites, α-ZOL has the highest capacity to bind ERs, followed by ZEA and β-ZOL [122].

Yip and collaborators reported that ZEA binds to ERs which are presented in different target cells and tissues including the immune cells and is able to modulate their expression [123]. ER-α expression was found in macrophages, B cells, NK cells, CD4+T cells, CD8+T cells, while ER-β expression is presented in monocytes, macrophages, B cells or NK cells [124,125]. The ratio between the two receptors ER-α and ER-β and their density is different in various cells and tissues [126]. The expression of ER-α after ZEA exposure was increased in the pig jejunal explants [25], but a decrease in the expression of this receptor and an increase in that of ER-β in the colon of intoxicated mice was observed [61].

ERs are members of the nuclear hormone receptors family that acts as transcription factors [127] which interact with chromatin target sites by two different path-ways: One dependent of estrogen response element (ERE) and another ERE-independent through signal transduction via RAS/mitogen-activated protein kinase (MAPK) and phosphoinositide 3-kinase (PI3K/Akt) [127,128]. After activation, ERs translocate into the nucleus where they form complexes with co-regulatory proteins, transcription factors, at specific DNA sites leading to epigenetic modifications of chromatin and to the initiation of the transcription process [129]. Very recently, it was demonstrated that ZEA induces ER translocation in endometrial stroma cells [130], but this was not proved yet for immune cells.

### 6.1. Mechanisms Triggered by ZEA in Proliferation of the Immune Cell

**Figure 2:**
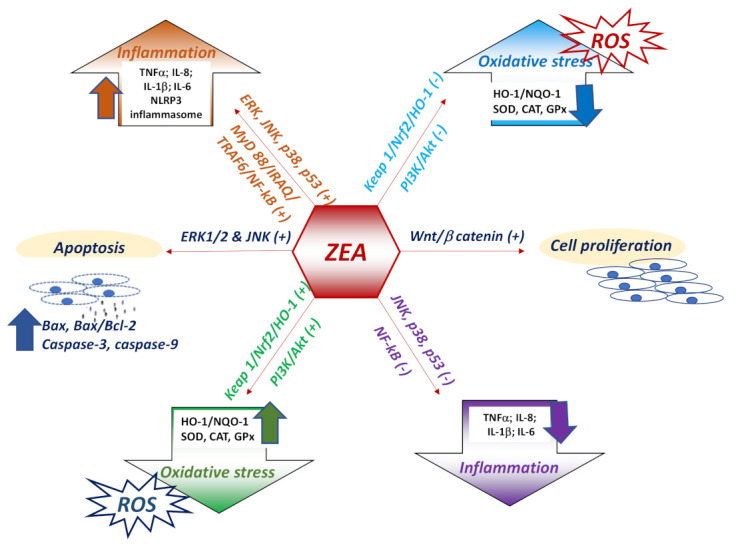
Suggested mechanisms involved by zearalenone in immune cells.

As already presented in the previous sections, ZEA can induce opposite biological effects in various cells/tissues that could be related to the ratio between ER-α vs. ER-β and the density of ER receptors. For example, it was shown that the activation of ER-α is related mainly to cell proliferation, while the activation of ER-β rather inhibits cell proliferation when it is co-expressed with ER-α [131]. In intestinal pig explants, activation of ER-α following ZEA exposure was associated with an activation of pro-proliferative Wnt/β catenin signaling pathway and a down regulation of immunosuppressive TGF-β signaling [25] (Figure 2).

In other immune tissues, zearalenone is responsible for a decrease of cell proliferation and apoptosis [29,38] as previously described. According to a recent review of Zeng and collaborators concerning the capacity of zearalenone to induce cell proliferation or to determine cell death, at least three mechanisms are involved: (i) Alteration of the cell cycle as well as of the expressions of cell cycle regulating proteins; (ii) cell oxidative stress and (iii) cell apoptosis through ERs stress, ROS producing and mitochondrial signaling pathway [132]. These mechanisms are equally involved in apoptosis and cell death induced by zearalenone in immune cells. ZEA induced apoptosis in T lymphocytes, increase the ratio of Bax/Bcl-2 and promote the expression of cleaved caspase-3 and cleaved caspase-9, which indicates that the toxin activates the mitochondrial apoptosis pathway [133]. Similarly, an increased number of apoptotic cells were observed in the Peyer’ patches of the 20 mg/kg b.w. ZEA-treated mice associated with a significantly increase of Bax gene expression and of the ratio of Bax:Bcl-2 [38].

In mouse MODE-K intestinal epithelial cells exposed to zearalenone, the toxin increased the gene expression and protein synthesis of molecules involved in ERS-induced apoptosis pathway as: c-Jun N-terminal kinase (JNK), C/EBP homologous protein, GRP78, caspase-1 and the pro-apoptotic protein Bax, while decreasing the levels of the anti-apoptotic related protein Bcl-2 [35].

The apoptosis triggered by the exposure of T lymphocytes to ZEA was accompanied by the over activation of MAPKs as extracellular regulated protein kinases (ERK) and JNK [133]. ERK pathway is involved in cell proliferation, differentiation and survival [134], while JNK is involved in apoptosis, inflammation, cytokine production, and metabolism [135]. The over activation of MAPKs following ZEA action was shown also in another cell types [119], but the mechanisms responsible for MAPKs activation is still unclear. It was suggested that for ERK1/2 activation, an important role is played by G-protein-coupled protein homolog, GPR30 associated or not with the estrogen receptor (α or β) [136].

### 6.2. Mechanisms Triggered by ZEA in Inflammation and Oxidative Stress

Many studies have linked apoptosis induced by zearalenone to oxidative stress [137,138,139]. For example, an in vitro study on RAW264.7 macrophage cells showed that cellular apoptosis is induced by ZEA through ROS accumulation, leading to the activation of p53-mediated signaling pathways, JNK and p38 MAPK-kinases. ZEA is thought to activate JNK and p38 MAPK, which drive to changes in mitochondrial Bcl-2/BAX proteins, a decrease of mitochondrial membrane potential, and ultimately to ROS generation. Through a self-propagation mechanism, increased ROS stimulates again JNK/p38 phosphorylation in ZEA-contaminated cells [93]. p38 MAPK signaling activation by the oxidative stress produced via cytochrome P450 reductase was found to be the main pathway through which ZEA induces toxicity in IPEC-J2 intestinal epithelial cells. It was observed that ZEA increased the level of both gene and phosphorylated protein expression of *p38 MAPK* which led to the autophagy induction [140]. The fact that ZEA affects the immune response via MAPK-kinases modulation is also suggested by the results obtained by Pistol et al., in the spleen of piglets receiving a diet contaminated with 316 ppm of ZEA in which JNK pathway was responsible for the activation of the inflammatory response assessed by pro-inflammatory cytokines and other molecules involved in inflammatory processes (MMPs/TIMPs) [141] (Figure 2).

Nuclear factor erythroid 2–related factor 2 (Nrf2) is an essential regulator of antioxidant genes, and it is early activated in response to the oxidative stress [142]. Nrf2-mediated pathway is considered one of the mechanism responsible for the alteration of the antioxidant and inflammatory responses induced by ZEA at least at the intestinal level [28,143]. The activation of the Keap1–Nrf2 signaling pathway was observed after ZEA exposure and might contribute to the reduction of ZEA toxicity in immune cells and organs [141,142]. However, other studies have shown that zearalenone compromise the capacity of the organism to provide an adequate response to the oxidative stress, resulting in a downregulation of Keap1/Nrf2/HO-1 pathway [144] and of phosphoinositide 3-kinase (PI3K)/Akt signaling pathway related to Nrf-2 [145].

Oxidative stress and inflammation are tightly correlated, reactive oxygen species playing an important role in the progression of inflammatory disorders [146] which are mainly regulated by the nuclear transcription factor-κB (NF-κB) pathway, the NF-κB receptor playing an essential role as a mediator in the regulation of the innate and adaptive immune responses [110]. An increase of Nrf2 after ZEA exposure was correlated with a decrease of NF-κB and the experimentally induction of NF-κB [22,141] significantly increased ZEA-induced oxidative stress [104]. However, the mechanisms by which zearalenone can influence the expression of the genes involved in the inflammatory response is unclear, zearalenone acting both as pro- or anti-inflammatory molecule/agent.

Although the involvement of ERS in the mechanism of apoptosis vs. cell death induced by ZEA is well defined, their involvement in the pro- or anti-inflammatory activity of ZEA is less evident. The activation of both ER-α or ER-β receptors reduced the inflammation [147,148] and this is the reason for which the estrogens are used as therapeutic agents in inflammation associated with metabolic diseases [149]. However, ER-α or ER-β were not involved in the alteration of the innate immune response induced by ZEA [51].

Toll like receptors (TLRs) plays a role in the ZEA induced inflammation. Pre-stimulation of RAW264.7 macrophages with different TLR ligands led to a different activation of TLR signaling and consequently to a different modulation of pro- and anti-inflammatory cytokines [38]. Thus, priming of RAW264.7 macrophages with TLR4 and TLR9 for 12h increase both expression and synthesis of IL-1β and decrease that of TNF-α, while the TLR2 priming resulted in increase of IL-1β, TNF-α and IL-10. Also, other studies showed that ZEA promotes a pro-inflammatory response in response to TLR ligand stimulation. Increased protein and mRNA expression of trans-membrane receptor TLR4 was observed in kidney of pregnant rats exposed to increased concentrations of zearalenone and was associated with an increase of NF-κB/p65 and pro- inflammatory cytokine level [150]. TLR4 plays an important role in initiating and accelerating of inflammation and it is a key element in the TLR4/MyD88 signaling pathway [151]. It can be assumed that TLR-4 role in ZEA induced inflammation is very important especially under simultaneously infectious disease and mycotoxin contamination. Indeed, using a microarray technology and the analyses of gene expression pattern it was observed that the exposure of IPEC-1 cells to 10µM of ZEA up-regulated the expression of several genes involved in the inflammatory response, while the co-exposure to both ZEA and *Escherichia coli*, up-regulated 33 genes [45]. Also, in the same cell line, co-exposure to ZEA and *E. coli* resulted in an increase of inflammatory response through an activation of the MyD88/IRAQ/TRAF6/NF-κB pathway via TLR4 [33]. In vivo studies demonstrated that at the level of the two important immune organs ZEA generate a biphasic inflammatory effect by triggering two different mechanisms. In the spleen of growing piglets fed diet contaminated with 316ppb of ZEA, 10% of the total up-regulated genes were genes involved in inflammation. The inflammatory mechanism involved by ZEA was the activation of JNK pathway [141]. Through mechanisms related also to MAPK-kinases pathways activation (ERK and p38), ZEA induced in activated neutrophiles the formation of neutrophil extracellular trap, consisting in extracellular chromatin filaments with a major role in inflammation [138]. By contrast, in the liver ZEA produced a dramatically decreased in gene expression coding for pro-inflammatory cytokines along with the decrease of NF-κB and p38 MAPK gene expression.

Recent studies have shown the involvement of inflammasome in the inflammation induced by ZEA. In vitro and in vivo exposure to ZEA activated the ROS-mediated NLRP3 inflammasome in mouse peritoneal macrophages and in the colon of ZEA exposed mice, which resulted in the caspase-1 dependent activation of the inflammatory cytokines IL-1ß and IL-18 [152]. NF-κB/p65 activation induced by ZEA also contribute to the activation of the NLRP3 inflammasome, inflammatory response and cell death caspase-1 dependent in insulinoma cell line [153]. However, in colon tissue of mice with dextran sulphate induced colitis ZEA was able to reduce the inflammatory reaction [154]. The immunosuppressive effect of ZEA is due to the inhibition of MAPK activity (TAK1/JNK/p38) and NF-κB [64].

The capacity of ZEA to interfere with the immune response is probably related to an alteration of the organism self-tolerance. For example, in pig spleen, ZEA can decrease the expression of FoxP3 gene, a master gene involved in the immune tolerance mechanisms that can be correlated with a potentiation of the inflammatory response [141]. This hypothesis is also sustained by a decrease of Treg cells after ZEA exposure with consequences on self-tolerance maintenance and control of the immune response [38].

In conclusion, despite an increasing number of studies concerning the mechanisms involved in immunotoxicity induced by ZEA, the available data are unsubstantial and needs further works. Nevertheless, the data presented here in showed that zearalenone and its metabolites are immunotoxic and altered the immune cell viability and proliferation, cell cycle as well as immune cells functionality such as inflammatory response and their capacity to synthesize active molecules. In this review, we have suggested the mechanisms responsible for the immunotoxic effects induced by ZEA. However, more studies are needed in the future, in order to validate the proposed mechanisms.

## Figures and Tables

**Table 1:** Effect of ZEA on the humoral immune response.

Effect (s)	Species/Cell Type	Dose/Time of ZEA Administration	References
↑ IgE, ↓ IgM no changes of IgG and IgA	BALB/c mice female	20 mg/kg b.w. 14 days	[38]
↓ IgG, IgA ↑ IgM	Pregnant rats	100,150 mg/kg feed 7 days	[70]
↓ IgG, IgM, IgE no changes of IgA	Wistar rats female	0, 1, 5, 30 mg/kg b.w. 36 days	[72]
↓ Ig A, Ig G	BALB/c mice, female	40 mg/kg b.w. 48h	[71]
↓ IgG ↑ Ig M	Wistar rats male	2 mg/kg b.w./week 3 weeks	[69]
↓ Ig G no changes of IgA, IgM	Prepubertal gilts	200, 800, 1600 μg/kg feed 14 days	[73]
↓ IgG, IgM no changes of IgA	Piglets male	0.8 mg/kg feed 4 weeks	[74]
↓ IgG, IgM in serum ↑ IgA in serum	Kunming mice, female	30 mg/kg b.w. 12 days	[75]
↓ IgG in serum no changes on IgA, IgM	post-weaning female piglets	1.1-3.2 mg/kg feed 18 days	[76]
no effect of serum IgG, IgM, IgA	B6C3F1 mice	10 mg/kg feed 6 weeks	[77]
↓ IgG, IgA, IgM in cell SN	Swine PBMC	10 mM 7 days	[19]
↓ IgG ↓ IgM	BALB/c mice	5, 10, 15 mg/kg b.w./day 2 weeks	[66]

**Table 2:** Humoral immunity with specific antibody/in vaccination.

Effect (s)	Species/Cell Type	Dose/Time of ZEA Administration	References
↓ B cells producing IgM to SRBC ^1^	Wistar rats female	3 mg/kg b.w. 28 days	[57]
No differences in humoral immune response against SRBC ^1^	prepubertal gilts	0.75 mg/kg feed 21 days	[81]
↓ Ab titer to porcine parvovirus	Wistar rats	5 mg/kg b.w. 36 days	[72]
↓ Ab titer to swine plague	post-weaning female piglets	1.1–3.2 mg/kg feed 18 days	[76]
No effect on the splenic PFC ^2^ response to SRBC ^1^ Delayed hypersensitivity response to keyhole limpet hemocyanin	B6C3F1 female mice	10 mg/kg b.w. 2–8 weeks	[79]

^1^ SRBC (sheep red blood cell); ^2^ PFC (plaque forming cells).

**Table 3:** Effect of ZEA on cellular immune response.

Effect (s)	Species/Cell Type	Dose/Time of ZEA Administration	References
↓ CD4^+^, CD8^+^, CD11c^+^ in spleen ↓ CD4^+^, CD8^+^, F4/80^+^ and ↑ CD19^+^ and CD11c^+^ in the mesenteric lymph nodes ↑TNFα and apoptosis, ↓ IL-6	BALB/c mice (female, 7-week-old)	5,20 mg/kg b.w.2 weeks	[38]
**ZEA 100 and 150 mg/kg:** ↓ viability of splenocytes ↓ T-cell proliferation Induce histopathological damage in spleen **ZEA 150mg/kg:** ↑ interleukin IL-6, IL-18 and IL-1β ↓ interferon-γ, TNFα and IL-10 in spleen	Sprague Dawley Pregnant Rats	50, 100, 150 mg/kg b.w. 7 days	[70]
disrupt the proliferation of CD4^+^8+ in peripheral blood cells	Polish Landrace x Polish Large White crossbreeds	0.5 mg/kg 6 weeks	[105]
↓ IL-1 in thymus and spleen ↓ IFN-γ in serum ↓ IL-2, IL-6, IL-10 in thymus ↓ IL-10 and IFN-γ in the spleen	Wistar rat	1, 5, 30 mg/kg b.w. 36 days	[72]
↓ CD3^+^, CD4^+^, CD8^+^, CD56^+^ cells	BALB/c mice	40 mg/kg b.w. 48h	[71]
↓ CD4^+^, CD8^+^ cells in peripheral blood	BPC and SPC Sheep	3.07– 14.49 μg/kg feed winter time	[106]
**ZEA 40 µM:** Inhibit T cell-chemotaxis by CCL19 ↑ CD4^+^ T cells induced by CCL19 chemotaxis **ZEA 20 µM:** ↑ CD8+ T cells induced by CCL21 chemotaxis ↓ expression of chemokine receptor CCR7 and CCR2	BALB/cmouse splenic lymphocytes	10, 20, 40 µM 48h	[107]
↓ CD3^+^CD4^+^ T cells ↑ CD3^+^CD8^+^ T cells	Female Kunming Mice	20, 30 mg/kg b.w. 12 days	[108]
↓ CD21^+^B, CD2^+^T, CD4^+^CD8^−^T ↑ CD8^+^CD4^−^ and TCRγδ^+^ T	Polish Large White female	0.1 mg/kg 42 days	[109]
↓ CD4^+^CD8^+^, CD4^+^, CD4^+^/CD8^+^(2 mg/kg) ↑ CD8^+^ (3.2 mg/kg)	Landrace × Yorkshire × Duroc Piglets	1.1, 2, 3.2 mg/kg feed 18 days	[76]
↑ IL-1β and IL-6, ↓ IFN-γ cytoplasmic edema chromatin deformation splenic damages	Yorkshire × Landrace × Duroc Piglets	1.1, 2.0, 3.2 mg/kg feed 18 days	[110]
↓ IFN-γ, IL-10↓ proliferation	kidneys of piglets	0.8 mg/kg 4 weeks	[111]
↑ IL-2, ↓ IL-6	Isa Brown chicken splenic lymphocytes	0.1–25 μg/mL 48 h	[82]

**Table 4:** The effect of zearalenone on immune organs weight and structure.

Effect (s)	Species/Cell Type	Dose/Time of ZEA Administration	References
Thymic atrophy with histological and thymocyte phenotype changes and decrease in the B cell percentage in the spleen	Wistar rats	3.0 mg/kg b.w. 28 days	[57]
Atrophy of white pulp and swelling of red pulp	post-weanling gilts	2.0, 3.2 mg/kg feed 18 days	[110]
No effect on spleen and bursa of Fabricius weights	one-day-old broiler chicks	10–800 mg/kg feed 21 days	[113]
No effect on spleen and bursa of Fabricius weights	one-day -old broiler chicks	50–800 mg/kg feed 3 weeks	[114]
Enlargement of the spleen in males	Sprague Dawley rats	1.25, 3.75 mg/kg b.w. 8 weeks	[115]
No effect on spleen weight	Sprague Dawley rats	0.5, 0.9, 1.8, 3.6 mg/kg b.w. 4 weeks	[116]
No effect on spleen weight	BALB/c mice female, 7-weeks-old	5, 20 mg/kg b.w. 14 days	[38]
No effect on spleen weight	White Leghorn female chickens, 2-weeks-old	50, 200, 400, 800 mg/kg b.w. 7 days	[117]
No macroscopic changes and no histopathologic effect on lymph nodes	32-day-old gilts	0.75 mg/kg feed 21 days	[81]
No effect on thymus and spleen weights No histopathologic changes	B6C3F1 weanling female mice	10 mg/kg feed 56 days	[77]
Decreased immune organ weight and lymphocyte counts, lymphoid atrophy and depletion in the spleen	BALB/c female mice	40, 80 mg ZEN/kg b.w. 28 days	[118]

## Data Availability

Not applicable.
